# Reduced Dynamic Complexity of BOLD Signals Differentiates Mild Cognitive Impairment From Normal Aging

**DOI:** 10.3389/fnagi.2020.00090

**Published:** 2020-04-08

**Authors:** Haixia Zheng, Keiichi Onoda, Atsushi Nagai, Shuhei Yamaguchi

**Affiliations:** ^1^Laureate Institute for Brain Research, Tulsa, OK, United States; ^2^Department of Neurology, Faculty of Medicine, Shimane University, Izumo, Japan

**Keywords:** mild cognitive impairment, multi-scale entropy, resting-state fMRI, BOLD complexity, imaging biomarker

## Abstract

Mild cognitive impairment (MCI) is characterized as a transitional phase between cognitive decline associated with normal aging and Alzheimer’s disease (AD). Resting-state functional magnetic resonance imaging (fMRI) measuring blood oxygenation level-dependent (BOLD) signals provides complementary information considered essential for understanding disease progression. Previous studies suggested that multi-scale entropy (MSE) analysis quantifying the complexity of BOLD signals is a novel and promising method for investigating neurodegeneration associated with cognitive decline in different stages of MCI. Therefore, the current study used MSE to explore the changes in the complexity of resting-state brain BOLD signals in patients with early MCI (EMCI) and late MCI (LMCI). We recruited 345 participants’ data from the Alzheimer’s Disease Neuroimaging Initiative database, including 176 normal control (NC) subjects, 87 patients with EMCI and 82 patients with LMCI. We observed a significant reduction of brain signal complexity toward regularity in the left fusiform gyrus region in the EMCI group and in the rostral anterior cingulate cortex in the LMCI group. Our results extend prior work by revealing that significant reductions of brain BOLD signal complexity can be detected in different stages of MCI independent of age, sex and regional atrophy. Notably, the reduction of BOLD signal complexity in the rostral anterior cingulate cortex was significantly associated with greater risk of progression to AD. The present study thus identified MSE as a potential imaging biomarker for the early diagnosis of pre-clinical Alzheimer’s disease and provides further insights into the neuropathology of cognitive decline in prodromal AD.

## Introduction

Mild cognitive impairment (MCI) is a condition in which individuals exhibit cognitive decline exceeding that expected with normal aging that does not notably interfere with activities of daily life (Petersen, 2004). However, previous evidence revealed that more than 50% of people with MCI progress to dementia, particularly Alzheimer’s disease (AD), within 5 years (Gauthier et al., 2006). Owing to lack of effective clinical treatment for AD, early detection of individuals with MCI is increasingly important to permit early and adequate clinical interventions to reduce the risk of progression to AD. Also, because the anatomical or functional brain changes in patients with MCI are extremely subtle and often overlap (Guerrero et al., 2014), it has been a major challenge to discriminate brain structural and functional alterations between patients with MCI and normal control (NC).

Resting-state functional magnetic resonance imaging (fMRI) studies based on blood oxygen level-dependent (BOLD) signal, which indirectly measures neural activity by detecting changes in blood oxygenation, have revealed the potential of BOLD signals as a biomarker for early detection of the neurophysiological alterations associated with MCI. This is attributable to its non-invasive and task-free nature (Ogawa et al., 1990; Deyoe et al., 1994), as well as because functional changes appear well before structural changes (Pievani et al., 2011; Teipel et al., 2015) and detectable cerebrospinal fluid amyloid-β and phosphorylated tau abnormalities (Chen et al., 2016). It has become evident that functional connectivity (FC) is altered in multiple brain regions such as the posterior cingulate cortex, thalamus, hippocampus, temporal gyrus and occipital gyrus in subjects with MCI (Weiner et al., 2017). Although FC and brain network analysis has provided valuable insights into differences between patients with MCI and NC subjects, the observations have not been consistent, and the mechanism underlying these alterations remains largely unknown. However, this linear statistical approach, which assesses the correlation of BOLD signals between brain areas, underestimates the complex and dynamic activity of the BOLD time series, thus limiting its capability and sensitivity for characterizing the complex human brain function alterations related to cognitive decline. Hence, it is critical to consider this issue while analyzing spontaneous fluctuations of resting-state BOLD signals.

Over the past few years, a non-linear statistical method named sample entropy has been introduced to study the complexity and regularity of physiological systems, as well as human brain function associated with aging and cognitive decline (Richman and Moorman, 2000; Yang et al., 2013; Smith et al., 2014). Sample entropy is a concept which originally came from information theory which proved a mathematical way of quantifying the complexity/irregularity of finite length time series (Pincus, 1991; Richman and Moorman, 2000). When applied to several coarse-sampled scales from the original time series, sample entropy can be extended to multi-scale entropy (MSE) to better capture the dynamic complexity of neural signals across multiple temporal scales (Costa et al., 2018). To date, evidence has suggested a general trend of decreasing complexity in EEG signals with aging and worse performance of memory-related tasks (Wang et al., 2016; Sheehan et al., 2018). Furthermore, both resting-state fNIRS and resting-state fMRI have observed reduced brain signal complexity in patients with AD compared with NC group across several networks, especially the default mode network (DMN) (Grieder et al., 2018; Li et al., 2018). These preliminary studies indicate that MSE can be a valid index for cognitive decline that better captures dynamic changes of BOLD signals than FC.

In the current study, we obtained BOLD rs-fMRI data from the Alzheimer’s Disease Neuroimaging Initiative (ADNI), applying a non-linear approach by using MSE to probe brain function changes among healthy subjects and patients with MCI. We anticipated lower MSE values in prodromal AD than in normal aging. Accumulating evidence indicates that the pathologies and functional abnormalities involved in the development of AD-associated cognitive deterioration are complex and vary by disease state (Lee et al., 2014; Teipel et al., 2016; Weiner et al., 2017; Fletcher et al., 2018). Therefore, in an effort to investigate pre-symptomatic AD progression at different stages, patients with MCI have been classified into two subgroups based upon clinical and behavioral measures provided by ADNI at the time of the imaging study: early MCI (EMCI) and late MCI (LMCI). This enabled the investigation of AD biomarkers and potential disease-related factors in various clinical stages, which is essential for uncovering AD developmental processes and designing effective early interventions to slow disease progression.

## Materials and Methods

### Subjects

The dataset used in this study was obtained from the ADNI database^1^. The ADNI was launched in 2003, and the primary goal has been to test whether imaging, other biological markers and clinical and neuropsychological assessments can be combined to measure the progression of early dementia^2^. Detailed inclusion criteria for the diagnostic categories can be found on the ADNI website^3^. Briefly, all subjects in this study aged between 55 and 90 years with Mini-Mental State Examination (MMSE) scores of 24–30 and geriatric depression scale of ≤5. Subjects with EMCI or LMCI had a subjective memory concern as identified by themselves or their partners, an abnormal Logical Memory II score (education-adjusted) and a clinical dementia rating (CDR) of 0.5 (memory box ≥0.5). The score ranges of Logical Memory II for EMCI were 9–11 for 16 years of education, 5–9 for 8–15 years and 3–6 for 0–7 years, and the ranges for LMCI were ≤8 for 16 years of education, 4–7 for 8–15 years and 2–3 for 0–7 years. In addition, the general cognition and functional performance of patients with EMCI or LMCI was sufficiently preserved such that a diagnosis of AD could not be made. NC subjects had no memory complaints beyond that expected by aging (verified by a partner), a normal Logical Memory II score (education-adjusted) and CDR = 0 (memory box = 0). In total, 101 patients with EMCI (73.3 years old, 52 females), 96 patients with LCMI (73.9 years old, 45 females) and 204 NC subjects (74.5 years old, 121 females) from the database were analyzed in this study. Data were selected according to the availability of resting-state fMRI datasets for patients with MCI and age-matched healthy subjects. Among these, longitudinal follow-up data for an average of 13 ± 20 months were also collected. The demographic information for the ADNI subjects is summarized in Table 1. There were no significant age and sex differences between the groups. The MMSE score was significantly lower in the EMCI and LMCI groups than in the NC group.

**TABLE 1:** Demographic data.

	**EMCI**	**LMCI**	**NC**	**statistics**
N (N of analysis)	101 (87)	96 (82)	204 (176)	
Age	73.3 ± 9.0	73.9 ± 7.1	74.5 ± 9.2	n.s.
Sex (female/male)	52/49	45/51	121/83	n.s.
MMSE	28.2 ± 1.8	27.9 ± 1.8	28.9 ± 1.5	EMCI, LMCI < NC
Conversion time (months) (N)	23.6 ± 16.3 (8)	25.3 ± 14.8 (13)	–	n.s.
Follow-up duration (months)	21.4 ± 24.6	13.7 ± 19.4	8.8 ± 17.2	n.s.

*The two-sample *t*-test for age, chi-squared test for sex and Kruskal–Wallis test for Mini-Mental State Exam (MMSE) were used. The values in parentheses for N indicate the numbers of subjects who had no excessive head movement (<4.5 mm). The values in parentheses for conversion time indicate the numbers of subjects who converted to Alzheimer’s disease. NC, normal control; EMCI, early mild cognitive impairment; LMCI, late mild cognitive impairment.*

### Image Acquisition

FMRI data were acquired using Philips 3T scanners. The measurement parameters of resting-state fMRI were as follows: repetition time (TR) = 3000 ms, echo time (TE) = 30 ms, slice thickness = 3.3 mm, voxel size = 3 mm × 3 mm × 3 mm, flip angle (FA) = 80°, slice number = 48 and 140–200 time points. The structural T1 images were obtained using the following parameters: TR = 6.72 ms, TE = 3.16 ms, slice number = 171, field of view: 256 mm × 256 mm, voxel size: 1.0 mm × 1.0 mm × 1.2 mm and FA = 9°.

### Pre-processing of Functional Images

Statistical Parametric Mapping (SPM12) and functional connectivity toolbox (CONN) (Whitfield-Gabrieli and Nieto-Castanon, 2012) were used for pre-processing. The functional images were re-aligned to remove any artifacts caused by head movement. Subjects who moved their heads excessively (more than 4.5 mm) were excluded from the following analysis (remaining numbers of subjects: 87 for EMCI, 82 for LMCI and 176 for NC). ANOVA confirmed there were no head movement differences among the three groups (EMCI, 1.7 ± 1.1 mm; LMCI, 1.4 ± 1.2 mm; NC, 1.5 ± 1.1 mm; *F* = 1.08, *p* = 0.34). The images were corrected for differences in image acquisition time between slices and were normalized to a Montreal Neurological Institute template space using the DARTEL method (re-sampled into 3 mm × 3 mm × 3 mm voxels). Spatial smoothing was applied with a full-width half-maximum (FWHM) equal to 8 mm. The linear trends of time courses were removed, and the effect of head movement parameters and mean BOLD signals from the whole brain, white matter and cerebrospinal fluid were removed at each voxel. Temporal filtering (0.01 Hz < f < 0.1 Hz) was applied. Finally, each voxel time series was standardized to a mean of zero and standard deviation of unity.

The analysis of the gray matter volume (GMV) was performed according to voxel-based morphometry (VBM) on Computational Anatomy Toolbox (CAT12^4^). The VBM procedure involves the segmentation of the original structural MRI images in gray matter, white matter and cerebrospinal fluid. The gray matter images were normalized to templates in stereotactic space and smoothed using a 6-mm FWHM Gaussian kernel. Gray matter mask was used to exclude voxels outside of the brain from further statistical analysis.

### MSE

**FIGURE 1:**
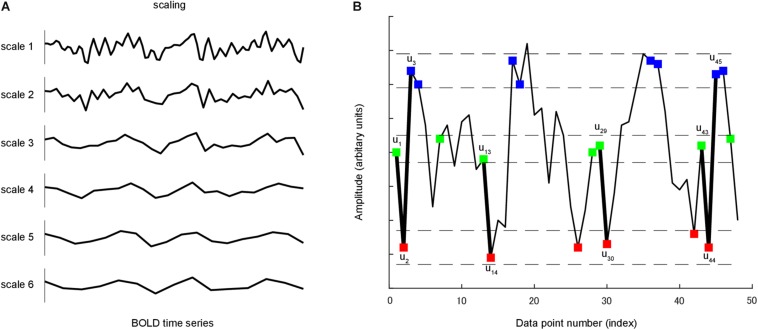
**(A)** A coarse-graining process was applied to time series. For a given time series, multiple coarse-grained time series were constructed by averaging the data within non-overlapping windows of increasing length. **(B)** A time series u is shown to illustrate the procedure for calculating sample entropy. In this case, the pattern length m is 2, and the similarity criterion r is 0.3 of the standard deviation of u. Dotted horizontal lines around u_1_, u_2_ and u_3_ represent u_1_ ± r, u_2_ ± r and u_3_ ± r, respectively. Two data values match each other, that is, they are not distinguishable, if the absolute difference between them is less than r. Green, red and blue points represent points that match u_1_, u_2_ and u_3_, respectively. Consider the m-component green-red template sequence (u_1_, u_2_) and (m + 1)-component green-red-blue (u_1_, u_2_, u_3_) template sequence. For the segment shown, three green-red sequences matched the template, whereas only one green-red-blue sequence matched the template. In this case, the numbers of sequences matching the two- and three-component template are 3 and 1, respectively. These calculations were repeated for the next two- and three-component template sequences. The numbers of sequences that matched each of the two and three components were added to the previous values. This procedure was repeated for all other possible template to determine the ratio between the total numbers of two- and three-component template matches. Sample entropy is the natural logarithm of this ratio.

Richman and Moorman (2000) proposed sample entropy measuring the complexity and regularity of clinical and experimental time series data (Figure 1). Sample entropy estimates the randomness or irregularity of a time series by determining how often different patterns of data are found in the given time series. It is defined by the negative natural logarithm of the conditional probability that a time series data of pattern length *m* (the sequence length of adjacent time points to be compared), having repeated itself within a tolerance of *r* (similarity factor), will also repeat itself for *m* + *1* time points without allowing self-matches (Richman and Moorman, 2000). A large value of sample entropy represents unpredictability or random variation (high complexity) whereas a small value represents predictability or a more regular structure (low complexity). MSE consists of a set of sample entropy values under multiple time scales. Therefore, MSE method incorporated two procedures: First, a “coarse-graining” process was applied to the time series. For instance, time series at scale 3 was constructed by averaging every 3 data points without overlapping. For time series scale 1, the coarse-grained time series is simply the original time series (Figure 1A). Second, sample entropy was calculated for each coarse-grained time series scale (Figure 1B).

The Complexity Toolbox^5^ was used to compute the MSE maps of resting-state fMRI data for each subject at each voxel. Pattern length *m*, distance threshold *r* and time scale *l* were set to calculate MSE. For short datasets (time series length, approximately 100), sample entropy for *r* ≥ 0.3 (in the case of *m* = 2) agreed with the theoretical value (Richman and Moorman, 2000). Previous fMRI studies used the sample entropy set *m* = 1 or 2 and *r* = 0.30–0.45 (Smith et al., 2014; Yang et al., 2015, 2018; Wang et al., 2018). In the current study, MSE was calculated for each BOLD time series based on *m* = 2 and *r* = 0.3 across the range of time scales from 1 to 6.

Group level analysis was conducted by using ANCOVA with age, gender and scan duration as covariates at both the global and voxel level. The statistical criteria were a false-discovery rate (FDR) corrected *p* < 0.05 at the cluster level and uncorrected *p* < 0.001 at the voxel level. Then, we extracted individual MSE data of a sphere radius of 6 mm with the peak voxel of a significant cluster as the centre, which was for the visualization and following analysis. To investigate the effect of brain atrophy on MSE, we conducted correlation analysis between the sample entropy and GMV. As performed for the MSE data, we extracted the GMV data from the same cluster of individual subjects.

### Cox Proportional Hazards Regression

The Cox proportional hazards regression model was used to analyze the relationship between brain entropy and time to convert to AD in consideration of censored data. For each individual, the event was considered conversion to AD. Survival time was defined as the interval between the month of MRI measurement at baseline and diagnosis at follow-up. In an effort to minimize the effect of age and gender, we used a linear regression model to regress out gender and the age at baseline scan (years). We then entered the residuals into the Cox regression model. An FDR adjusted *p* value < 0.05 was considered to indicate a statistical significance. Additionally, the difference of the risk of AD conversion between high entropy and low entropy groups was estimated using the Kaplan-Meier method with the log-rank test. The high-low group assignment was based on a median-split threshold. The analysis was performed using RStudio version 1.1.463 (Integrated Development for R. RStudio, Inc., Boston, MA, United States. URL^6^).

### Data Availability

The datasets analyzed for this study can be found in the Alzheimer’s Disease Neuroimaging Initiative (ADNI) database (adni.loni.usc.edu).

## Results

**FIGURE 2:**
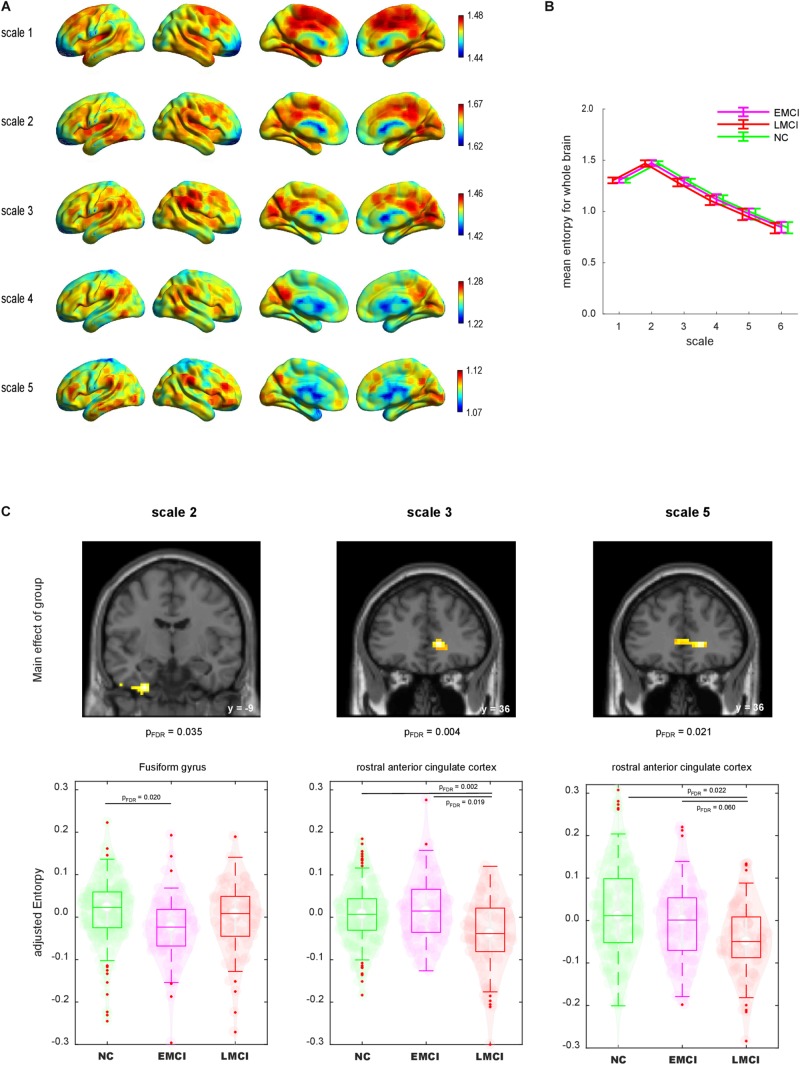
**(A)** Lateral and medial views of mean maps of sample entropy for scales 1–6. The value range was different for each scale. **(B)** Mean sample entropy of the whole brain for scales 1–6. Error bar denotes standard deviation. **(C)** Group comparisons of sample entropy using whole-brain ANCOVA. Top: Brain regions displayed significant main effects of group (voxel level *p* < 0.001 and cluster level false-discovery rate corrected *p* < 0.05). Bottom: Violin plot of each individual sample entropy value. Boxes indicate the mean and interquartile. Whiskers indicate the interquartile range. NC, normal control; EMCI, early mild cognitive impairment; LMCI, late mild cognitive impairment.

Figure 2A shows the lateral and medial views of mean MSE across groups for each scale. For scale 1 entropy value was generally higher at the middle cingulate cortex, supplementary motor cortex and posterior medial prefrontal cortex than in other brain regions. For scales 2 to 5, the temporo-parietal junction, lateral and medial prefrontal cortex, posterior cingulate cortex and primary visual cortex exhibited a relatively higher entropy value than other brain regions. Statistical group difference comparison was carried out at global and voxel level:

First, we calculated the global mean sample entropy of the whole brain for each subject and plotted it by group (Figure 2B). The global mean sample entropy was highest at scale 2, and it gradually decreased along with the increase of scales. ANCOVA with age, gender and scan duration as covariates was conducted to test whether global mean sample entropy differs among groups. There were no significant global mean sample entropy differences among the groups at any scale.

Next, we compared the entropy map of each scale among the groups at the voxel level. We found significant differences among the groups regarding the sample entropy at scales 2, 3, and 5 (Figure 2C). The analysis revealed that the left fusiform gyrus (FG) at scale 2 exhibited a significant main effect of group [(x, y, z) = (−27, −9, −42), voxel size = 49, FDR corrected *p* = 0.035]. To illustrate the group difference, we extracted the individual sample entropy of voxels within a sphere (*r* = 6 mm) with the peak as a centre and calculated the averages. The scatter plot indicated that the sample entropy was lower for the EMCI group than for the NC group. The decrease of sample entropy in the LMCI group was not significant in direct comparison. At scales 3 and 5, the rostral anterior cingulate cortex (rACC) displayed significant main effects of group [scale 2: (15, 36, 3), size = 82, FDR corrected *p* = 0.004; scale 5: (18, 36, 0), size = 65, FDR corrected *p* = 0.021]. The sample entropy of the rACC was decreased in the LMCI group, whereas the EMCI group did not display a significant decrement.

**FIGURE 3:**
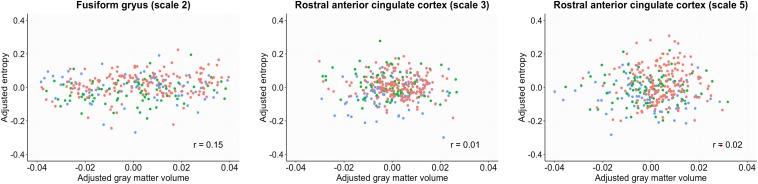
Scatter plots between sample entropy and gray matter volume in the fusiform gyrus and rostral anterior cingulate cortex. The gray matter volume was estimated by regressing out age, gender and total intracranial volume. The entropy was estimated by regressing out age and gender. Red, normal control; Green, early mild cognitive impairment; Blue, late mild cognitive impairment.

The GMV was estimated by regressing out age, gender and total intracranial volume. The entropy was estimated by regressing out age and gender. GMV in the FG and rACC did not show significant group differences (*p* > 0.07). The correlation analysis revealed no significant correlation coefficient between the sample entropy and GMV in the FG and rACC (*rs* < 0.15, *p* > 0.58, Figure 3).

To assess the association between these reductions in brain local BOLD signal entropy and time to progress to AD, we conducted Cox proportional hazards regression analysis. Local BOLD signal entropy was adjusted for age and gender by regressing out age and gender using a linear regression model. We found a negative association between entropy in the rACC at scale 5 and time to convert to AD with a beta coefficient of −7.11 (FDR corrected *p* = 0.009). This association was confirmed in all samples as well as the LMCI group (beta coefficient = −8.21, FDR corrected *p* = 0.048), which indicated that a smaller value of entropy in the rACC was associated with a significantly greater risk of progressing into AD. There were no statistically significant associations between entropy in other brain regions and time to convert into AD (see detailed results in Table 2).

**TABLE 2:** Risk factors associated with conversion to Alzheimer’s disease after adjustment using the Cox proportional hazards regression model.

	**All samples**				**LMCI**				**EMCI**			
**RF**	**BC**	**SE**	**HR**	**P**	**BC**	**SE**	**HR**	**P**	**BC**	**SE**	**HR**	**P**
FG_S2	−1.98	2.13	0.14	0.35	−0.09	3.11	1.09	0.98	−2.31	4.13	0.10	0.58
rACC_S3	−3.29	2.96	0.04	0.27	−0.27	4.03	0.76	0.95	−1.93	4.73	0.15	0.68
rACC_S5	−7.11	2.42	0.0008	0.009**	−8.21	3.83	0.0003	0.048*	−2.73	3.60	0.07	0.45

All of the risk factor estimates were the residuals from a linear regression model which regressed out age and gender. RF, risk factor; BC, beta coefficient; SE, standard error; HR, Hazard ratio; FG_S2, entropy in the fusiform gyrus at scale 2; rACC_S3, entropy in the rostral anterior cingulate cortex at scale 3; rACC_S5, entropy in the rostral anterior cingulate cortex at scale 5; EMCI, early mild cognitive impairment; LMCI, late mild cognitive impairment; *FDR adjusted *p* < 0.05, **FDR adjusted *p* < 0.01.

**FIGURE 4:**
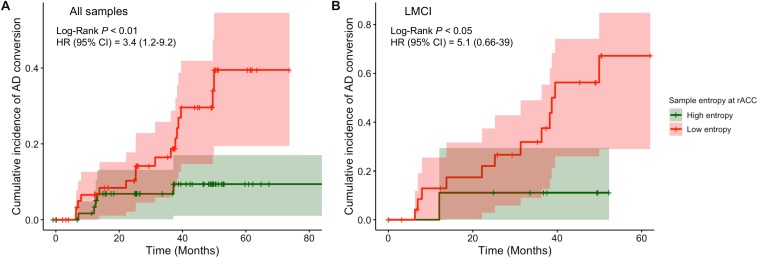
Comparison of cumulative incidence of AD conversion under condition of high entropy and low entropy at rostral anterior cingulate cortex. **(A)** All samples were included. **(B)** Only LMCI subjects were included. AD, Alzheimer dementia; HR, hazard ratio; CI, confidence intervals.

To better capture the risk of reduced entropy in rACC converting to AD, we divided subjects into high and low entropy groups by using a median-split approach and estimated the difference of the risk of AD between groups. Subjects with lower BOLD signal entropy at rACC scale 5 were associated with a 3.4 times (adjusted hazard ratio was 3.4 with a 95% confidence interval of 1.2 to 9.2, *p* < 0.01, Figure 4A) increased risk of converting to AD compared with the high entropy group. This risk was 5.1 times if the subjects were in LMCI group (adjusted hazard ratio was 5.1 with a 95% confidence interval of 0.66 to 39, *p* < 0.05, Figure 4B). There was no significant difference in the EMCI group.

## Discussion

The results of the current analyses reveal that people with EMCI or LMCI exhibit reduced resting-state fMRI BOLD signal complexity toward the regular pattern compared with the NC group. In the EMCI group, reduced complexity was observed in the FG, whereas in the LMCI group, this reduction was found in the rACC. These alterations were not caused by gray matter atrophy. More importantly, these reductions of brain signal variability in the rACC region exceeded structural atrophy, and it was associated with a greater risk of progressing into AD.

Our findings are consistent with the literature in this area. Previous studies demonstrated a general trend of decreasing signal complexity with normal aging and cognitive decline associated with AD (Yang et al., 2013; McIntosh et al., 2014; Grieder et al., 2018; Li et al., 2018; Sheehan et al., 2018). A few study used resting-state fNIRS/fMRI to investigate neural signal complexity among NC, MCI and AD groups (Wang et al., 2017; Li et al., 2018). Consistent with our findings, a reduction of BOLD signal complexity in the FG was observed in patients with AD compared with the findings in the MCI or NC group (Wang et al., 2017). The FG is located in the ventral portion of the temporal lobe and under the parahippocampal gyrus. Although the function of the FG is unclear, it has been associated with object recognition and face perception (Baroni et al., 2017; Weiner et al., 2018). Probably limited by small sample sizes and their methods of measuring entropy that considered only the ranks of the samples and not their metrics (Bandt and Pompe, 2002), previous studies failed to find meaningful alterations in the EMCI or LMCI group compared with the NC group. Our study therefore filled this gap by using larger sample sizes and sample entropy to provide new evidence by using MSE to assess different stages of MCI. The findings of reduced BOLD signal intensity in different regions between EMCI and LMCI suggests that BOLD signal complexity changes associated with cognitive decline are stage-dependent and are sensitive for detecting very early stages of prodromal AD.

Previous studies consistently observed lower brain signal complexity in DMN nodes in patients with AD compared with the NC subjects (Wang et al., 2017; Grieder et al., 2018; Li et al., 2018). Interestingly, we observed a trend toward decreased BOLD signal complexity with increasing cognitive decline in the rACC, which is a key component of the DMN. Although this finding did not reach significance in the EMCI group, it appears that BOLD signal variability decreases with the progression of AD. This hypothesis was supported by our finding of a correlation between the reduction of BOLD signal complexity in the rACC with the risk of progressing to AD via Cox proportional hazards regression analysis. Anatomically, the rACC has strong connections with both “emotional” limbic systems such as the anterior insular cortex and amygdala and “cognitive” systems such as the posterior cingulate cortex, prefrontal cortex and striatal brain regions (Etkin et al., 2006; Palomero-Gallagher et al., 2008; Stevens et al., 2011). Functionally, the rACC has been demonstrated to play a key role in the regulation of cognitive influences on emotional processing (Stevens et al., 2011; Szekely et al., 2017), especially when performing emotional conflict regulation tasks (Etkin et al., 2006, 2011). Moreover, strong functional connectivity between the ACC and other brain regions such as the hippocampus, frontal gyrus and temporal gyrus has been associated with excellent memory capacity in older adults, even in the presence of amyloid deposition (Lin et al., 2017). Therefore, our finding of significant MSE reduction in the rACC region in the LMCI group compared with the data in the NC group suggests that cognitive decline might be associated with disturbances of cognitive-emotional interactions in the brain. It has long been observed that progressive atrophy and glucose hypometabolism in the medial temporal lobe and cingulate cortex were associated with pre-symptomatic stages of AD (Fox et al., 2001; Knopman et al., 2013; Teipel et al., 2016). Together with the observations of a strong association of glucose metabolism with BOLD signals (Tomasi et al., 2013; Zhang et al., 2013), our evidence highlighted the involvement of rACC function in the neuropathology of cognitive decline in prodromal AD.

This study had a few potential limitations. First, previous studies suggested that the ADNI MCI criteria have a high rate of false-positive diagnostic errors (Bondi et al., 2014). This might explain why the reduction of rACC signal complexity in the EMCI group did not reach statistical significance. Further studies of MSE using MCI samples are warranted to determine if these findings are pathophysiologically relevant. Second, we evaluated cross-group and follow-up data; however, these data were incomplete because of subject dropouts or other factors such as study design and cost constraints. Although the multivariate Cox proportional hazards regression model considers censored data, future studies with well-designed longitudinal data are needed to support our interpretation. Third, MSE is a mathematical measure of BOLD signal complexity at the voxel level. The current study and existing evidence offer very limited insight into the association between macroscopic alterations and underlying biology in the context of cognitive decline. Future work combined with other methods such as dynamic functional connectivity or fluorodeoxyglucose positron emission tomography imaging is warranted.

In sum, our study indicated that significant reductions of brain BOLD signal complexity can be detected in patients with EMCI or LMCI. These alterations were independent of age or sex, and significantly associated with greater risk of AD conversion. Our findings indicate that MSE based on resting-state BOLD signals is more sensitive than functional connectivity or structural MRI; therefore, it could represent a quantitative functional marker for cognitive decline that may be useful for early screening in older adults at risk for AD.

## Data Availability Statement

The datasets analyzed for this study can be found in the Alzheimer’s Disease Neuroimaging Initiative (ADNI) database (adni.loni.usc.edu).

## Ethics Statement

The studies involving human participants were reviewed and approved by the Shimane University Ethics Committee. The patients/participants provided their written informed consent to participate in this study.

## Author Contributions

HZ, KO, AN, and SY conceived and designed the research. HZ and KO performed research. HZ and KO analyzed the data. HZ and KO wrote the manuscript with input from AN. All authors reviewed the manuscript. SY approved the final submission.

## Conflict of Interest

The authors declare that data used in preparation of this article were obtained from the Alzheimer’s Disease Neuroimaging Initiative (ADNI) database (adni.loni.usc.edu). As such, the funder and the investigators within ADNI contributed to the data collection, but did not participate in analysis, interpretation of data, the writing of this article or the decision to submit it for publication.

## Footnotes

1 adni.loni.usc.edu

2 www.adni-info.org for up-to-date information

3 http://adni.loni.usc.edu/methods/

4 http://dbm.neuro.uni-jena.de/cat/

5 http://loft-lab.org/index-5.html

6 http://www.rstudio.com
